# An In Vitro Study regarding the Wear of Composite Materials Following the Use of Dental Bleaching Protocols

**DOI:** 10.3390/jfb14100532

**Published:** 2023-10-21

**Authors:** Alexandru Dan Popescu, Mihaela Jana Ţuculină, Lelia Mihaela Gheorghiță, Andrei Osman, Claudiu Nicolicescu, Smaranda Adelina Bugălă, Mihaela Ionescu, Jaqueline Abdul-Razzak, Oana Andreea Diaconu, Bogdan Dimitriu

**Affiliations:** 1 Department of Endodontics, Faculty of Dental Medicine, University of Medicine and Pharmacy of Craiova, 200349 Craiova, Romania; alexandrudanpopescu20@gmail.com (A.D.P.); leliagheorghita@yahoo.com (L.M.G.); preda.smaranda@yahoo.com (S.A.B.); oanamihailescu76@yahoo.com (O.A.D.); 2 Department of Anatomy and Embriology, Faculty of Dental Medicine, University of Medicine and Pharmacy of Craiova, 200349 Craiova, Romania; andreiosman3@gmail.com; 3 Department ENT, Clinical Emergency County Hospital of Craiova, 200642 Craiova, Romania; 4 Department of Engineering and Management of the Technological Systems 1 Calugareni, Faculty of Mechanics, University of Craiova, 220153 Drobeta-Turnu Severin, Romania; 5 Department of Medical Informatics and Biostatistics, Faculty of Dental Medicine, University of Medicine and Pharmacy of Craiova, 200349 Craiova, Romania; mihaela.ionescu@umfcv.ro; 6 Department of Infant Care–Pediatrics–Neonatology & Doctoral School, University of Medicine and Pharmacy of Craiova, 200349 Craiova, Romania; jaquelineabdulrazzak90@gmail.com; 7 Department of Endodontics, Faculty of Dentistry, University of Medicine and Pharmacy Carol Davila Bucharest, 050474 Bucharest, Romania; bogdan.dimitriu@umcfcd.ro

**Keywords:** dental wear, dental bleaching, dental restoration, composite resin

## Abstract

Composite materials used in dental restorations are considered resistant, long-lasting and aesthetic. As the wear of restorations is an important element in long-term use, the aim of this study was to evaluate the surface condition of nanohybrid and microfilled composite resins, after being subjected to the erosive action of dental bleaching protocols. This paper reflects a comparative study between one nanofilled composite and three microfilled composites used in restorations. For each composite, three sets of samples (under the form of composite discs) were created: a control group, an “office bleach” group with discs bleached with 40% hydrogen peroxide gel, and a “home bleach” group with discs bleached with 16% carbamide peroxide gel. Wear was numerically determined as the trace and the coefficients of friction obtained using a tribometer, the ball-on-disk test method, and two balls: alumina and sapphire. For all composite groups, there were statistically significant differences between the wear corresponding to the control and bleaching groups, for both testing balls. Regarding the composite type, the largest traces were recorded for GC Gradia direct anterior, for all groups, using the alumina ball. In contrast, for the sapphire ball, 3M ESPE Filtek Z550 was characterized by the largest traces. With respect to the friction coefficients, the “office bleach” group recorded the largest values, no matter the composite or the ball type used. The 3M ESPE Valux Plus composite recorded the largest friction coefficients for the alumina ball, and 3M ESPE Filtek Z550 for the sapphire ball. Overall, the “office bleach” group was characterized by higher composite wear, compared to the “home bleach” protocol or control group. Nanofilled composite resins showed superior wear resistance to microfilled resins after undergoing a bleaching protocol.

## 1. Introduction

Dental composites are some of the most widely used dental restorative materials in modern dental practice. The demand for these dental materials is continuously increasing; this is due to interdisciplinary collaborations, as the properties of dental composites are being improved every day [1,2].

Dental restorations using composite materials are highly appreciated by both dentists and patients due to their excellent aesthetic, mechanical and chemical properties. Teeth restored with dental composites perform their functions normally in the dento-maxillary apparatus, notably resisting to occlusal forces [3,4,5].

The mechanical properties of composite materials depend very much on the type of filler and especially on the particle size of the filler. Dental composites could be divided into microfilled, macrofilled, hybrid, modern hybrid and nanocomposites benefiting from a multitude of filler particles of different sizes [6,7].

Macrofilled composites have particle sizes ranging from 0.1 μm to 5 or even 6 μm, while microfilled composites benefit from much smaller particle sizes (0.01–0.05 μm). The higher the particle concentration and the smaller the size of the filler, the stronger the composite material will be [8,9,10].

The emergence of nanocomposites on the market has been an extremely important moment in modern dental practice. They are frequently used as dental restorative materials due to their excellent mechanical properties, stability over time, resistance to bending and, last but not least, their superior aesthetics [11,12,13].

Depending on the size of the constituent particles in the filler, nanocomposites can be nanohybrid or nanofilled. Nanofilled dental composites contain nanometric particles, while nanohybrid dental composites contain both micrometric and nanometric particles in their fillers [14,15].

Due to the increasing demands of physiognomic appearance, the clinical procedure of tooth bleaching is experiencing great popularity among patients. Among the most commonly used tooth-bleaching agents, hydrogen peroxide in various concentrations (30–40%) is notable, while carbamide peroxide is found in lower concentrations (10–16%) [16,17,18].

The use of tooth-bleaching agents is performed with great care, respecting the bleaching protocols. The main tooth-bleaching techniques are “in-office” tooth bleaching which generally uses 35% hydrogen peroxide while “at home” tooth bleaching uses 10% carbamide peroxide [19,20].

During clinical tooth-bleaching procedures, both hydrogen peroxide and carbamide peroxide come into contact with various dental restorative materials. Following dental bleaching protocols, there is a possibility that the mechanical or chemical properties of dental restorations may be affected [21].

The surface quality of dental restorative materials can be modified following clinical tooth-bleaching procedures. There are situations where the surface roughness and porosity of dental restorative materials increase following the application of bleaching agents. On the other hand, the microhardness of dental restorative materials decreases following the application of bleaching protocols [22,23,24].

All the mechanical properties of dental composites are closely interrelated, and the microhardness of the surface of dental restorative materials can influence their wear. A decrease in the surface microhardness of composites can lead to a decrease in the wear resistance of these dental materials [25,26].

Composite wear is a particularly important factor in the success and durability of dental restorations. In addition to the mechanical or chemical properties of dental restorative materials, environmental or mechanical factors in the oral cavity can influence their durability [26,27].

In restorations, longevity, aesthetics, and long-term clinical success are directly related to the wear of the restorative materials. The aim of this study was to evaluate the surface condition of nanohybrid and microfilled composite resins, after being subjected to the erosive action of dental bleaching protocols, considering as null hypothesis the fact that there is no significant change after the use of bleaching gels.

## 2. Materials and Methods

For this study, four composite materials were selected, as presented in Table 1.

### 2.1. Composite Discs

For each composite listed in Table 1, a series of test samples were selected, appropriately labeled, and documented. A stainless-steel tool was used to create the samples in the form of composite discs, with dimensions of 10 ± 0.1 mm and 2.5 ± 0.1 mm (the maximum layer thickness permitted by each composite manufacturer for shade A2). Composite materials were put in the apparatus to create the samples. The samples were made by applying finger pressure to remove excess resin. Two glass plates were used to create a level surface by applying compression on both sides, and to ensure consistency and air-bubble removal. The final samples had a thickness of 2.4 mm, plus or minus 0.1 mm. The samples were measured using a Mitutoyo digital micrometer IP65 (Mitutoyo Corporation, Kawasaki, Japan). The previously defined samples’ dimensions were correctly achieved, as the device had the capacity to apply and condense the composite in a single layer.

Following the manufacturer’s recommendations, a clinically applicable experimental protocol was adopted. The photopolymerization duration was set in accordance with each manufacturer’s specifications. Using the LED E. Woodpecker wireless lamp (Guilin Woodpecker Medical Instrument Co., LTD., Guilin, China) with a light intensity of 700 mW/cm^2^, photopolymerization was performed successively for 20 s above the higher placed plate and for 20 s below the lower plate (for a total of 40 s). Bidirectional photopolymerization was employed to ensure that the process was accurate and thorough, because the two glass plates were used to maintain the same distance from the composite material. This process limited any potential photopolymerization impact on the results, as the glass plate’s thickness was used as a benchmark to consistently maintain the lamp’s distance from the samples.

The samples were finished and polished using the Super-Snap X-Treme Kit (Shofu Dental Corporation, San Marcos, TX, USA) after being kept in distilled water at a temperature of 37 °C for 24 h. Each sample was finished and polished by a single dentist, in accordance with the manufacturer’s instructions at standard revolutions per minute (RPMs).

### 2.2. Bleaching Guidelines

Each chosen material (GC G-aenial anterior, GC Gradia direct anterior, 3M ESPE Filtek Z550, and 3M ESPE Valux Plus) was represented by 15 discs, with a total of 60 discs for all composite types: 5 control samples, 5 samples subjected to “office bleach”, and 5 samples subjected to the “home bleach” method.

In the control group, composite disc samples were not treated with any bleaching agent. The second type included discs made of the same material as the control type, on which the dental bleaching procedure known as “office bleach” was administered in two 20 min treatments, using a coating of 40% hydrogen peroxide gel (Opalescence Boost PF 40% hydrogen peroxide, Ultradent, South Jordan, UT, USA) that was put in a 1 mm thickness. The samples were rinsed with a saline solution containing NaCl 0.9% after each application. Finally, they were kept at a temperature of 37 °C in containers filled with a saline solution of NaCl 0.9%.

The last set of samples consisted of discs made of the same composite materials as the control group, on which the “home bleach” dental bleaching procedure was performed using a layer of 1 mm of 16% carbamide peroxide gel (Opalescence Melon PF 16% carbamide peroxide, Ultradent, South Jordan, UT, USA), six hours a day for seven days. The samples were kept in a saline solution of NaCl 0.9% at 37 °C inbetween bleaching sessions.

### 2.3. Wear Measurement

A tribometer TRB 01-02541 (CSM Instruments, Peseux, Switzerland) was used to determine the friction coefficients. The parameters used were the following: ball-on-disk test method, linear test type, amplitude 3 mm, maximum linear velocity 6 cm/s, normal force 3 N, number of cycles 20,000 (120 m), acquisition rate 10.0 Hz. An alumina ball with roughness (Ra = 5.9 μm) and a sapphire ball with roughness (Ra = 3.9 μm) (balls’ diameters equal to 6 mm) were used for testing, the working temperature was 37 °C, and the working atmosphere was air. We used artificial saliva as a lubricant to simulate the physiological conditions found in the oral cavity, and the software used was InstrumX Software (2.5A, CSM Instruments, Peseux, Switzerland) (operating system of the computer—Microsoft Windows 2000 XP).

The samples were fixed, one at a time, in the tribometer vice and artificial saliva was applied to their surface. The ball (either alumina or sapphire) was fixed in the tribometer rod and the two discs representing the normal force of 3 N (2 N + 1 N) were added to the holder. The test parameters were entered into the corresponding program and then a similar test was performed for all 60 samples.

**Figure 1:**
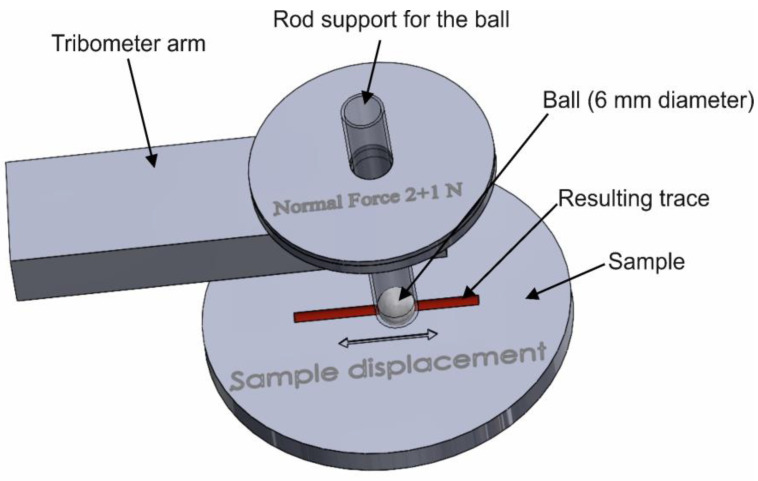
Schematic process of wear testing.

The schematic process of wear testing is presented in Figure 1.

After determining the friction coefficients, resulting traces were studied using a NIKON MA 100 optical microscope, equipped with NIS ELEMENTS BR software (4.5, Laboratory Imaging, Prague, Czech Republic) for data acquisition and processing.

#### Specific Wear Coefficients Analysis

For samples with concave traces, the specific wear coefficients *K* were calculated with the following equation:

(1) $$K=\frac{W}{F\cdot L}\;[{mm}^{3}/N\cdot mm]$$

where *K* is the specific wear coefficient, *W* is the wear volume [mm^3^], *F* is the testing force [N], *L* is the total sliding distance [mm].

For samples with convex traces, the worn cross-sectional areas could not be determined, because the material adhered to the sample’s surface (adhesion wear) and there was no depth within the traces, as in the case of samples with concave traces (abrasion wear).

### 2.4. Scanning Electron Microscopy Analysis

SEM (Scanning Electron Microscopy, Thermo Fisher Scientific, Eindhoven, The Netherlands) analysis of the samples was performed using the Phenom Pure Pro X electronic microscope (Thermo Fisher Scientific, Eindhoven, The Netherlands) and Phenom Pro Suite acquisition software (2015, Thermo Fisher Scientific, Eindhoven, The Netherlands). This device integrates an optical microscope with 20× optical zoom and an electronic microscope with a magnification range of 70–30,000×.

Samples were prepared according to the following protocol: Impurities were removed from the samples’ surface using a compressed air can, then a conductive double-sided tape was glued to a supportive device (which is cylindrical, with a diameter of 10 mm). After each sample was glued to the device, it was inserted in a charge reduction sample holder, which will be placed in the microscope. This equipment has the following advantage: for a non-metallic sample, it reduces the electron charge; therefore, the preparation of nonconductive samples is no longer necessary. After the device with the sample is inserted, the image of the sample is displayed, and the areas of the SEM are defined. For this analysis, the microscope settings were the following: intensity of electron cannon 10 keV, magnification 320x, and working distance 3 mm under the cylindrical device.

### 2.5. Statistical Analysis

Tooth wear parameters were statistically processed using the software application Statistical Package for Social Sciences (SPSS), version 20 (IBM Corp., Armonk, NY, USA). Continuous parameters were expressed as mean ± standard deviation (SD) or medians. Group comparisons were performed using one-way ANOVA for normally distributed data, followed by Tukey or Games–Howell for post hoc analysis, or the Kruskal–Wallis test followed by Dunn’s procedure for post hoc analysis, in all other cases. The value *p* < 0.05 was considered statistically significant, as the threshold α was set to 5%.

## 3. Results

**Figure 2:**
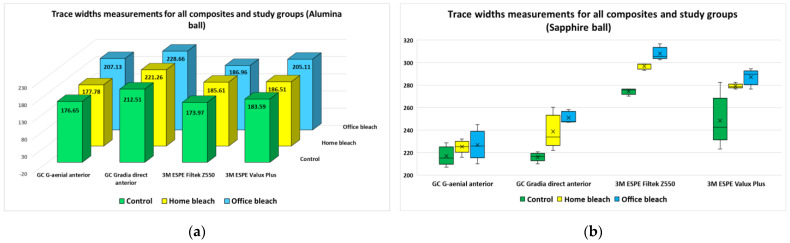
Trace width measurements for each composite, study group and ball type: (**a**) alumina ball; (**b**) sapphire ball.

For trace widths, the average or median values of the five measurements performed for each composite, study group and ball type, are emphasized in Figure 2a (alumina ball) and Figure 2b (sapphire ball). The measurements performed using the alumina ball were normally distributed within each sample set, for all composites, while the measurements performed using the sapphire ball were not normally distributed.

For both ceramic ball types, the discs included in the “office bleach” group had the largest width traces, followed by the “home bleach” group and the control group.

For the “office bleach” group, which reflected the highest values of trace width, the composite discs had very different behaviors, according to the specific ball type. The ESPE Filtek Z550 composite presented the lowest mean value of the trace width for the alumina ball, but the highest median value of the trace width for the sapphire ball. For the other three composites, the trace widths for the sapphire ball were higher compared to the trace widths for the alumina ball, indicating that the effect on the discs was more pronounced. Similar results were obtained for the “home bleach” and control groups, as the ESPE Filtek Z550 composite had extreme values, and overall, the alumina ball produced smaller trace widths than the sapphire ball.

Regarding the friction coefficients, for both ball types, the discs included in the “office bleach” group had the largest measured values, followed by the “home bleach” group and the control group.

**Figure 3:**
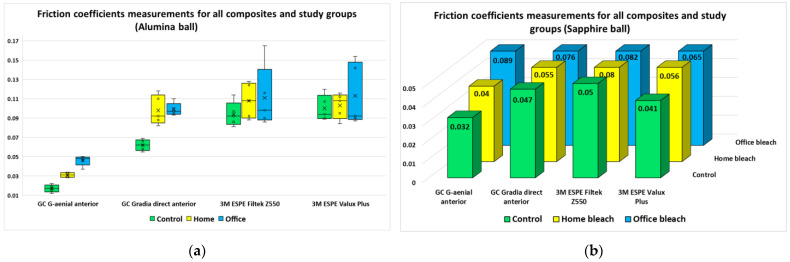
Friction coefficient measurements for each composite, study group and ball type: (**a**) alumina ball; (**b**) sapphire ball.

The mean/median values of the five measurements performed for each composite, study group and ball type are emphasized in Figure 3. The measurements performed using the alumina ball were not normally distributed within each sample set, for all composites, while the measurements performed using the sapphire ball were normally distributed.

### 3.1. Alumina Ball Analysis

#### 3.1.1. Composite Measurements for Alumina Ball

For each composite type, discs were classified into three groups: control (*n* = 5), “home bleach” (*n* = 5) and “office bleach” (*n* = 5).

A one-way ANOVA was conducted to determine if the width trace was different for groups defined by the bleach type. There were no outliers, and the data were normally distributed for each group, as assessed by a boxplot and Shapiro–Wilk test (*p* > 0.05), respectively. There was homogeneity of variances, as assessed by Levene’s test for equality of variances (*p* = 0.498 for GC G-aenial anterior, *p* = 0.122 for GC G-Gradia direct anterior, *p* = 0.144 for 3M ESPE Filtek Z550, and *p* = 0.168 for 3M ESPE Valux Plus). For all composites, the width trace increased from the control group, to the “home bleach” group, to “office bleach” groups, in this order, and the differences between groups were statistically significant (Table 2).

Friction coefficient measurements for each composite type were used as input data for a Kruskal–Wallis test conducted to determine if there were differences in trace width between the three groups (Table 2). Distributions of the friction coefficient values were similar for all groups, as assessed by the visual inspection of the boxplot. Median measurements were statistically significantly different between the different sample sets for GC G-aenial anterior and GC Gradia direct anterior, but not for the other composites.

As there are three groups (control, “office bleach” and “home bleach”), subsequent pairwise comparisons were performed using Tukey post hoc analysis for trace widths, and Dunn’s (1964) procedure with a Bonferroni correction for multiple comparisons, made with statistical significance accepted at the *p* < 0.0166 level (unadjusted significance threshold) for the friction coefficients. The statistically significant comparisons between groups are identified in Table 3.

#### 3.1.2. Bleaching Protocol Measurements for Alumina Ball

For each protocol, discs were classified into four groups with five measurements each, one for each composite type: GC G-aenial, GC Gradia, ESPE Filtek and ESPE Valux.

A one-way ANOVA was conducted to determine if the width trace was different for each bleaching protocol, for groups defined by the composite type (Table 4). There were no outliers for these data sets, and the values were normally distributed for each group, as assessed by boxplots and by the Shapiro–Wilk test (*p* > 0.05), respectively. There was homogeneity of variance, as assessed by Levene’s test for equality of variances for the control group (*p* = 0.106) and for the “office bleach” group (*p* = 0.066). For the “home bleach” group, the result of Levene’s test was *p* = 0.024, so there was no homogeneity of variances (therefore, the result is expressed based on Welch ANOVA). For all sample sets (control, “office bleach” and “home bleach”), the differences in width traces between composites were statistically significant (Table 4).

Friction coefficient measurements were used as input data for a Kruskal–Wallis test conducted to determine if there were differences between the three groups (Table 4). Distributions of friction coefficients values were similar for all groups, as assessed by the visual inspection of the boxplot. Median measurements were statistically significantly different between the different composites for all sample groups (Table 4).

Since there were four groups, pairwise comparisons were performed using Tuckey or Games–Howell post hoc analysis for trace widths, and Dunn’s (1964) procedure with a Bonferroni correction for multiple comparisons with statistical significance accepted at the *p* < 0.0166 level (unadjusted significance threshold) for friction coefficients. The statistically significant comparisons between composites within each sample group are identified in Table 5.

### 3.2. Sapphire Ball Analysis

#### 3.2.1. Composite Measurements for Sapphire Ball

For each composite type, discs were classified into three groups: control (*n* = 5), “home bleach” (*n* = 5) and “office bleach” (*n* = 5).

Trace width measurements were statistically analyzed using the Kruskal–Wallis test, conducted to determine if there were differences between the three groups. Distributions of trace width values were similar for all groups, as assessed by the visual inspection of several boxplots. Median trace width measurements were statistically significantly different between the sample sets for all the composites, except GC G-aenial anterior (Table 6).

For the friction coefficients, a one-way ANOVA was conducted to determine if the friction coefficients were different for groups defined by the bleach type. There were no outliers, and the data were normally distributed for each group, as assessed by a boxplot and Shapiro–Wilk test (*p* > 0.05). There was homogeneity of variance, as assessed by Levene’s test: *p* = 0.450 for the GC G-aenial anterior composite, *p* = 0.948 for the GC G-Gradia anterior composite, *p* = 0.164 for the 3M ESPE Filtek Z550 composite, and *p* = 0.699 for the 3M ESPE Valux Plus composite. Mean friction coefficients were statistically significantly different between the different protocols, for all composites (Table 6).

As there were three groups, pairwise comparisons were performed using Dunn’s (1964) procedure, with the Bonferroni correction for multiple comparisons, made with statistical significance accepted at the *p* < 0.0166 level (unadjusted significance threshold) for trace widths, and Tuckey post hoc analysis for friction coefficients. The statistically significant comparisons between groups are identified in Table 7.

#### 3.2.2. Bleaching Protocols Measurements for Sapphire Ball

For each control and protocol (“office bleach” and “home bleach”) sample set, discs were classified into four groups with five measurements each, one for each composite type: GC G-aenial, GC Gradia, ESPE Filtek and ESPE Valux.

For each composite type, trace width measurements were used as input data for a Kruskal–Wallis test, conducted to determine if there were differences in trace widths between the three groups: “control” (*n* = 5), “home bleach” (*n* = 5) and “office bleach” (*n* = 5). Distributions of trace width values were similar for all groups, as assessed by the visual inspection of the boxplot. Median measurements were statistically significantly different between the different composites for all sample groups (Table 8).

A one-way ANOVA was conducted to determine if the friction coefficients were different for each bleaching protocols, for groups defined by the composite type. For each protocol, discs were classified into four groups with five measurements, one for each composite type: GC G-aenial, GC Gradia, ESPE Filtek and ESPE Valux. There were no outliers for these data sets, and the values were normally distributed for each group, as assessed by a boxplot and by the Shapiro–Wilk test (*p* > 0.05). There was homogeneity of variance, as assessed by Levene’s test for equality of variances for the “home bleach” group (*p* = 0.551) and for the “office bleach” group (*p* = 0.641). For the control group, the result of Levene’s test was *p* = 0.028, so there was no homogeneity of variance (therefore, the result is expressed based on the Welch ANOVA). For all sample sets (control, “office bleach” and “home bleach”), the differences in friction coefficients between composites were statistically significant (Table 8).

Since there were four groups, pairwise comparisons were performed using Tuckey or Games–Howell post hoc analysis for trace widths. For friction coefficients, we used Dunn’s (1964) procedure with a Bonferroni correction for multiple comparisons, with statistical significance accepted at the *p* < 0.0166 level (unadjusted significance threshold). For friction coefficients, pairwise comparisons were performed using Tuckey or Games–Howell post hoc analysis. The statistically significant comparisons between composites within each sample group are identified in Table 9.

### 3.3. SEM and Optical Microscopy Results

Following the analysis of the traces resulted after friction wear tests, Optical microscopy and SEM indicated that the traces’ surfaces were convex or concave.

Convex profile traces (deposits on the testing surface—wear by adhesion of own material) were obtained with the alumina ball for the following samples: GC G-aenial anterior control, GC Gradia direct anterior office bleach, GC Gradia direct anterior home bleach, 3M ESPE Filtek Z550 control, 3M ESPE Filtek Z550 office bleach, 3M ESPE Valux Plus control, 3M ESPE Valux Plus office bleach, and 3M ESPE Valux Plus home bleach. Convex profile traces were obtained with the sapphire ball for the following samples: GC G-aenial anterior control, GC G-aenial anterior office bleach, GC G-aenial anterior home bleach, GC Gradia direct anterior control, GC Gradia direct anterior office bleach, GC Gradia direct anterior home bleach, 3M ESPE Filtek Z550 control, 3M ESPE Valux Plus office bleach, and 3M ESPE Valux Plus home bleach.

**Figure 4:**
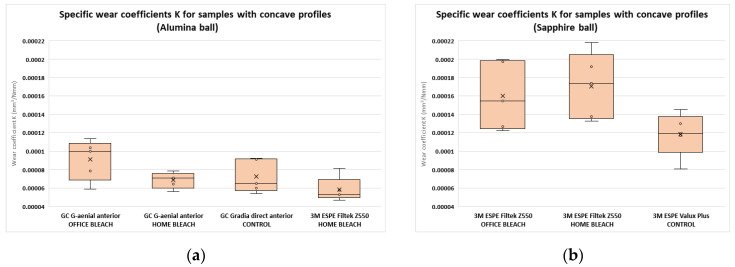
Specific wear coefficients K for samples with concave profiles: (**a**) alumina ball; (**b**) sapphire ball.

Concave profile traces (no deposits on the testing surface—wear by abrasion) were obtained with the alumina ball for the following samples: GC G-aenial anterior office bleach, GC G-aenial anterior home bleach, GC Gradia direct anterior control, and 3M ESPE Filtek Z550 home bleach. Concave profile traces were obtained with the sapphire ball for the following samples: 3M ESPE Filtek Z550 office bleach, 3M ESPE Filtek Z550 home bleach, and 3M ESPE Valux Plus control. The specific wear coefficients K were computed for all five composite discs included in each of the groups previously mentioned. As indicated in Figure 4 (4a for the alumina ball, and 4b for the sapphire ball), the specific wear coefficients computed following the measurements with the sapphire ball were greater than the coefficients computed following the measurements with the alumina ball.

Abrasion wear appeared for all samples, no matter the ball type (alumina or sapphire) but, in some cases, this phenomenon was followed by an adhesion process of the sample’s own material on its surface. For samples with convex profiles, the abrasion wear appeared at the beginning of testing, then it was followed by an adhesion of material in the worn area. Samples with concave profiles were characterized by abrasion wear, while the adhesion of material was insignificant compared to the other samples.

SEM images certify the fact that the traces’ surfaces presented humps corresponding to the adhesion or abrasion processes.

**Figure 5:**
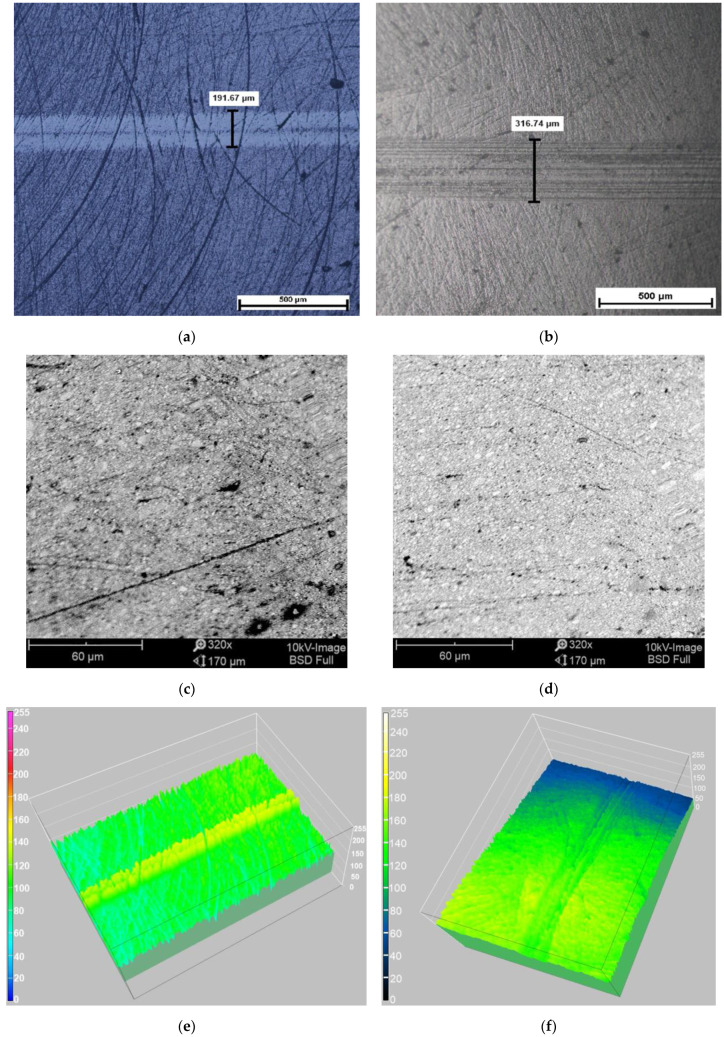
Trace width measurement for the 3M ESPE Filtek Z550 composite, after the “office bleach” protocol: (**a**) optical image of the trace after alumina-ball testing; (**b**) optical image of the trace after sapphire-ball testing; (**c**) SEM image of the trace after alumina-ball testing; (**d**) SEM image of the trace after sapphire-ball testing; (**e**) 3D image of the trace width, alumina ball; and (**f**) 3D image of the trace width, sapphire ball.

Figure 5a–f emphasize the trace widths following the testing process, with both alumina and sapphire balls.

## 4. Discussion

The null hypothesis of the study was that the wear of the materials does not change considerably after the acid attack of the bleaching gels, and the research hypothesis was that the surface condition of the materials changes significantly. This research hypothesis was primarily based on several previous studies proving that composite resin restorative materials can undergo degradation processes such as wear of the organic component of the material by its dissolution, and exposure of inorganic particles as a result of being subjected to acidic conditions [25,28,29,30].

In our study, 20 samples underwent “home bleach” and 20 samples were bleached using the “office bleach” protocol. The results showed that the surface wear values were higher for each of the four materials subjected to the bleaching protocols, compared to the control group, and the differences were statistically significant.

Regarding the four types of composites used in our study, several previous papers have proposed that their resistance to wear is influenced by the type, size, and degree of the inorganic particle loading. In the present study, microfilled composite resins provided lower surface wear values compared to nanohybrid composite resins for trace widths, for the sapphire ball. The specific wear coefficient K was also higher for the sapphire ball, compared to the alumina ball, for samples with concave profile traces, indicating a higher wear rate. This may be due to stresses occurring at the filler–resin matrix interface, with a possible loss of particles from the filler, and the exposure of the resin matrix susceptible to wear [31,32,33]. Other studies stated that composite resins with smaller filler particles have higher wear resistance [34,35].

The clinical success of a composite dental restoration also depends on its resistance to wear and fracture. Surface wear or fatigue is the result of contacts occurring during movement and sliding. Localized stresses occur as a result of direct contact between occlusal surfaces and are transmitted slightly below the surface, and are considered an important factor in the development of surface fatigue. This fatigue initiates the development of cracks in the substrate of restorative materials that over time can lead to extensive surface damage, generating material wear [36].

The lower modulus of elasticity of bulk-fill resins offers the advantage of reducing the shrinkage stress occurring during light-curing, but at the same time leads to more deformation and wear due to fatigue occurring both on the surface of the restorative material and on the structure of the tooth being restored with that material. These deformations are related to the size of the dental restoration, i.e., the larger the restorations, the more deformations, and the higher the water absorption of the composite, the more the dental restoration material is prone to wear. Therefore, the wear of bulk-fill composites was higher than that of other restorative materials [37,38].

Recent studies have concluded that the wear of bulk-fill composites is higher than that of conventional nanohybrid composites; bulk-fill restorative material can work satisfactorily for small restorations but not for large restorations or those placed in occlusal stress areas because wear of the material occurs [39].

A very important role in the wear resistance of composite materials is played by the percentage of filler and its particle size. The higher the filler content, the lower the risk of wear. Although the wear of composite resins is mainly affected by the material and filler properties, the composition of the monomers in the resin composition influences the wear of the composite, which leads us to conclude that wear is a complex and multifactorial process. An ideal dental restorative material should have physical and mechanical properties like those of the dental hard structure, and therefore the wear resistance should also be similar [7,40,41].

Nanohybrid composite resins contain a significant percentage of filler, and the particles in the filler’s constitution are nanometer-sized. These composites have good mechanical properties, less shrinkage during light curing, improved surface characteristics, higher homogeneity in the resin mass and due to the smaller particle size of the filler, a larger contact area with the organic matrix is provided [42].

During the mastication process, the rigid filler particles transmit mastication forces to the more elastic organic matrix of the composite resins. This can increase the stress at the interface between the filler and the organic matrix, which can lead to displacement of the filler particles and exposure of the more vulnerable organic matrix, resulting in material wear. Clinical studies performed on posterior tooth restorations found no significant difference in the wear of conventional resins compared to nanohybrid resins, while in vitro studies reported that nanohybrid composite resins may exhibit higher wear resistance [43,44].

In our study, nanofilled composite resins showed superior wear resistance to microfilled resins after undergoing a bleaching protocol, following the test performed with an alumina ball.

The amplitude of a composite wear as a function of time is represented by the wear rate, which is affected by factors such as the type of loading, temperature, type of movement, surface lubrication and, last but not least, the type of composite material [45,46].

Tooth wear by attrition is a physiological process as a result of tooth-to-tooth contact. This wear occurs in the absence of an abrasive agent and is influenced by the presence of saliva acting as a lubricant. The wear of composite resin dental restoratives is related to many factors, the most important of which are the particle size of the filler and the chemical formula of the resin matrix. Smaller filler particles have smaller spacing and less wear. The larger the particles, the lower the wear resistance of the composite resin. Of course, the bond between the filler and the composite resin matrix is another important factor involved in the wear of these dental restorative materials [41,47,48].

In our study, abrasion wear of dental composites depends on the microhardness of food particles that intervene in the mastication process, and on the elasticity and flexibility of composite resins used in restorations. The adhesion wear of dental composites is smaller compared to abrasion wear, and it may not be considered as a significant wear mechanism of dental composite resins.

Multiple studies have compared the wear of different types of composites; however, the results differ. Some studies report that the wear resistance of bulk-fill composite resins is similar to that of microfilled or nanohybrid composite resins, while other researchers have reported the higher wear of bulk-fill resins compared to conventional resins [49,50,51].

There are studies that have concluded that even roughness parameters determined at the surface of composite resins increased after wear testing. This is explained because wear exposed the particles in the composite resin filler and subsequently increased the surface roughness of the dental restorative material [52,53,54,55].

Prepolymerized materials in the composite resin filler do not silanise easily, cannot create strong bonds between particles and will not integrate easily into the resin matrix, which can lead to wear and thus increase the surface roughness of the composite resin. Methacrylate-based composite resins and their derivatives exhibit better gloss, surface roughness and lower wear than non-methacrylate containing composite resins, the latter having higher biocompatibility [56].

It has been shown that even tooth brushing can influence the surface roughness of composite resins and the gloss of these dental restorative materials. Tooth brushing causes abrasive wear on the surface of different types of composite resins, leading to an increase in roughness parameters and a decrease in gloss. As a result of the weak bonding between the large particles in the resin filling, some may detach from the surface of the composite resin due to the abrasion generated by brushing [57,58].

Wear due to abrasion by tooth brushing influences both the physical, mechanical, and optical properties of composite-resin dental restorative materials, impacting the aesthetics, hardness and roughness of composite resins [59,60].

The smoother a dental restoration is, the better its wear resistance and marginal integrity, thus increasing the life of the restoration and thus the oral health of the patient [60,61].

Therefore, the surface of a dental restorative material is important because it influences the wear of both the restorative material and the tooth, and the accumulation of bacterial plaque and tartar [62].

Jonne Oja et al. conducted a study in 2021 in which they performed the accelerated aging of microfilled and nanohybrid composite resins. They concluded that accelerated aging significantly reduced the bending properties of all composite resins studied compared to control samples. Other mechanical properties of dental restorative materials, such as microhardness and surface wear, were also studied, but accelerated aging did not produce a significant impact on the microhardness and surface wear of microfilled and nanohybrid composite resins [63,64,65,66].

In the case of composite resin restorations, the clinical data collected on wear show that the patient contributes much more than the material itself. Differences will always occur in the results obtained from testing composite resins in the laboratory versus those that are followed clinically [67].

In the oral cavity, teeth act like a tribological system, undergoing friction, lubrication (with saliva) and wear during masticatory functions. Over time, tooth surfaces undergo processes of degradation, trauma and carious damage, leading to loss of tooth tissue. Not only are teeth subject to the forces described above, but also dental restorative materials are subject to friction, lubrication, and wear [68,69,70,71,72].

The roughness of composite resins used in dental restorations is influenced by particle size while microhardness is influenced by the particle concentration of the filler. The addition of alumina and silicate particles had a positive effect on Young’s modulus and the wear resistance of composite resins [73,74,75,76,77].

A phenomenon of adhesion of the samples’ material to the balls’ surface (for both alumina and sapphire balls) may be observed during testing at the samples’ surface level, besides the wear process through adhesion and abrasion, respectively. Also, the corresponding traces are parallel with the direction of friction, but their widths are different. This effect was observed by other researchers too, while testing the surface of samples made of Ti_6_Al_4_V, Ti_6_Al_4_V–ZrO_2_ and ZrO_2_, following the direct contact with an alumina ball [78].

The values of the friction coefficients increase at the beginning of the testing process, then decrease, then they have a constant evolution over all the testing distance. This phenomenon was also tested by Bartolomeu et al. and it may be explained by the abrasion of the material after the contact with the alumina ball [79].

The differences between the friction coefficients may be explained by the chemical and physical interaction of dental composites with artificial saliva. The presence of artificial saliva plays an important role in the friction and wear of the dental composites. The same effect was observed by S. Madeira et al. when testing the interactions between ceramic materials and an alumina ball [80,81]. During the sliding contact, saliva acts like a lubricant and reduces the friction coefficient between hard surfaces.

During testing, the scraps of material form a solid/paste lubricant together with the artificial saliva, and this reduces the contact area, implicitly decreasing the friction coefficient values [80,81,82,83].

Rojas et al. tested the wear process with zirconia samples applied on natural enamel. Nowadays, zirconia represents an excellent physiognomic alternative to indirect teeth restorations; still, there are differences regarding the value of microhardness, compared to the one of natural enamel, which, in time, may lead to the wear of the natural tooth [83,84,85,86]. The value of zirconia’s microhardness (approximately 1430 HV) is superior to the microhardness of the enamel (approximately 410 HV), or to the one of dental composites (approximately 200 HV). Therefore, in our study, the alumina ball was chosen as it has an inferior microhardness value (1250 HV), and the sapphire ball was chosen as it has a superior microhardness value (1600 HV).

The shape of the wear varies between different types of wear, in the case of our study, between abrasion and adhesion wear, but also in the different stages of the same trace. The changes observed on the surface of the composite were flat, convex and concave, also showing striations and cracks.

SEM and optical microscopy studies can be useful for specifying the wear changes of the composite surface.

Recent studies have shown that length and depth of the scratches reveal a person’s dietary pattern. Additionally, in contrast to erosion, the depth/breadth ratio in abrasion tends to be steady over time [87,88,89].

The study of the influence of bleaching protocols on the surface of composite restorations opens new perspectives and leads to precise indications as to the type of material used in certain clinical situations. Achieving a smooth, wear-resistant surface is a very important goal for direct composite restorations. The accuracy of this clinical step influences the longevity of a restoration.

From a clinical point of view, the impact of food or prosthetic work made of various materials often makes the surfaces of restorations subject to uncontrollable wear force.

The novelty of the present study resides in the identification of dental wear areas, which are larger in cases of obturations already affected by chemical wear and are produced with items with greater microhardness (alumina and sapphire balls), while most studies used zirconia or other types of materials.

To our knowledge, in the past 20 years, there have been no published papers which have used items with a microhardness higher than that of zirconia.

The limitations of this study are represented by the small number of samples, the lack of similar studies whose results could have been compared to ours, and the lack of standardized forces applied to the sample surface, as well as the greater diversity of materials impacting the sample surface.

## 5. Conclusions

Composite materials undergo wear changes through adhesion and abrasion depending on the microhardness of the food particles involved in the mastication process, as well as the elasticity and flexibility of the composite resins used in restorations.

The tooth-bleaching protocol makes the surface of the composites more vulnerable to food impact.

## Figures and Tables

**Table 1:** Composite materials.

Name of Composite (Producer)	Type of Composite	Composite Matrix	Fillers Content (%) Wag
G-aenial Anterior (GC Tokyo, Japan)	Microfilled	UDMA ^1^, dimethacrylate comonomers	Prepolymerized silica and strontium fluoride (76 wt%).
Gradia Direct Anterior (GC Tokyo, Japan)		UDMA ^1^, dimethacrylates, trimethacrylates	Silica and prepolymerized resin (73 wt%)
Valux Plus (3M ESPE, Center St. Paul, MN, USA)		BIS-GMA ^1^, TEGDMA ^1^	Zirconia/silica (66 wt%)
Filtek Z550 (3M ESPE, Center St. Paul, MN, USA)	Nanohybrid	BIS-GMA ^1^, TEGDMA ^1^, UDMA ^1^, BIS-EMA ^1^, PEGDMA ^1^	Silica-zirconia, non-agglomerated/non-aggregated silica particles (82 wt%)

^1^ UDMA: Urethane Dimethacrylate. BIS-GMA: Bisphenol A-Glycidyl Methacrylate. TEGDMA: Tri-ethylene Glycol Dimethacrylate. BIS-EMA: Bisphenol A-Ethoxylated Dimethacrylate. PEGDMA: Polyethylene Glycol Dimethacrylate.

**Table 2:** Trace widths and friction coefficient measurements for all composites and protocols, alumina ball.

Composite	GC G-Aenial Anterior		GC Gradia Direct Anterior		3M ESPE Filtek Z550		3M ESPE Valux Plus	
Trace Width								
Group	Trace width (mean ± SD)	F(2,12)/ *p* *	Trace width (mean ± SD)	F(2,12)/ *p* *	Trace width (mean ± SD)	F(2,12)/ *p* *	Trace width (mean ± SD)	F(2,12)/ *p* *
Control	176.65 ± 20.72	5.625/ 0.019 ^#^	212.51 ± 11.77	4.085/ 0.044 ^#^	173.97 ± 5.56	9.149/ 0.004 ^#^	183.59 ± 5.82	34.002/ 0.0005 ^#^
Home Bleach	177.78 ± 16.92		221.26 ± 8.08		185.61 ± 3.55		186.51 ± 3.18	
Office Bleach	207.13 ± 8.97		228.66 ± 6.01		186.96 ± 6.32		205.11 ± 4.01	
Friction Coefficient								
Group	Friction coeff (median)	χ^2^(2)/ *p* **	Friction coeff (median)	χ^2^(2)/ *p* **	Friction coeff (median)	χ^2^(2)/ *p* **	Friction coeff (median)	χ^2^(2)/ *p* **
Control	0.017	12.500/ 0.002 ^#^	0.062	9.572/ 0.008 ^#^	0.092	1.902/ 0.386	0.094	0.185/ 0.911
Home Bleach	0.048		0.092		0.108		0.108	
Office Bleach	0.031		0.097		0.098		0.092	

* One-way ANOVA. ** Kruskal–Wallis H test. ^#^ statistically significant.

**Table 3:** Trace widths and friction coefficient variation between groups, for all composites and protocols, alumina ball—multiple group comparisons.

Composite	GC G-Aenial Anterior		GC Gradia Direct Anterior		3M ESPE Filtek Z550		3M ESPE Valux Plus	
Trace Width								
Group comparisons	Variation	*p* *	Variation	*p* *	Variation	*p* *	Variation	*p* *
Control—Home	30.48	0.030 ^#^	-	> 0.05	11.640	0.012 ^#^	-	> 0.05
Home—Office	29.350	0.036 ^#^	-	> 0.05	-	> 0.05	18.60	< 0.0005 ^#^
Control—Office	-	> 0.05	16.15	0.036 ^#^	12.990	0.006 ^#^	21.52	< 0.0005 ^#^
Friction Coefficient								
Group comparisons	Test statistic	*p* **	Test statistic	*p* **	Test statistic	*p* **	Test statistic	*p* **
Control—Home	-	> 0.05	−6.90	0.044 ^#^	Not applicable		Not applicable	
Home—Office	-	> 0.05	-	> 0.05				
Control—Office	−10.0	0.001 ^#^	1.20	0.012 ^#^				

* Tukey post hoc analysis. ** Dunn’s (1964) procedure with a Bonferroni correction. ^#^ statistically significant (adjusted significance for friction coefficient).

**Table 4:** Trace widths and friction coefficients measurements for all composites and protocols, alumina ball.

Composite	Control		Home Bleach		Office Bleach	
Trace Width						
Group	Trace width (mean ± SD)	F(3,16)/ *p* *	Trace width (mean ± SD)	F(3,8.263) **/ *p* *	Trace width (mean ± SD)	F(3,16)/ *p* *
GC G-aenial anterior	176.65 ± 20.72	9.889/ 0.001 ^#^	177.78 ± 16.92	25.659/ < 0.0005 ^#^	207.13 ± 8.97	33.769/ < 0.0005 ^#^
GC Gradia direct anterior	212.51 ± 11.77		221.26 ± 8.08		228.66 ± 6.00	
3M ESPE Filtek Z550	173.97 ± 5.56		185.61 ± 3.55		186.96 ± 6.32	
3M ESPE Valux Plus	183.68 ± 5.82		186.51 ± 3.18		205.96 ± 16.32	
Friction Coefficient						
Group	Friction coeff (median)	χ^2^(3)/ *p* ***	Friction coeff (median)	χ^2^(3)/ *p* ***	Friction coeff (median)	χ^2^(3)/ *p* ***
GC G-aenial anterior	0.048	16.211/ 0.001 ^#^	0.031	11.263/ 0.010 ^#^	0.017	10.782/ 0.013 ^#^
GC Gradia direct anterior	0.097		0.092		0.062	
3M ESPE Filtek Z550	0.098		0.108		0.092	
3M ESPE Valux Plus	0.092		0.108		0.094	

* One-Way ANOVA. ** Welch’s F. *** Kruskal–Wallis H test. ^#^ statistically significant.

**Table 5:** Trace widths and friction coefficient variation between composites for all protocols, alumina ball—multiple group comparisons.

Composite	Control		Home Bleach		Office Bleach	
Trace Width						
Group comparisons	Variation ^##^	*p* *	Variation ^##^	*p* **	Variation ^##^	*p* *
G-aenial—GC Gradia	13.10–58.62	0.002 ^#^	14.019–72.940	0.009 ^#^	14.019–72.940	0.009 ^#^
G-aenial—Filtek Z550	-	> 0.05	-	> 0.05	-	> 0.05
G-aenial—Valux Plus	-	> 0.05	-	> 0.05	9.643–33.416	< 0.0005 ^#^
Gradia—Filtek Z550	15.778–61.309	0.001 ^#^	21.576–49.723	0.001 ^#^	-	> 0.05
Gradia—Valux Plus	6.158–51.681	0.011 ^#^	20.641–48.858	0.001 ^#^	-	> 0.05
Filtek Z550—Valux Plus	-	> 0.05	-	> 0.05	0.0930–0.0247	< 0.0005 ^#^
G-aenial—Valux Plus	-	> 0.05	-	> 0.05		
Friction Coefficient						
Group comparisons	Test statistic	*p* ***	Test statistic	*p* ***	Test statistic	*p* ***
G-aenial—GC Gradia	-	> 0.05	-	> 0.05	−10.40	0.033 ^#^
G-aenial—Filtek Z550	−11.80	0.010 ^#^	−11.30	0.015 ^#^	−10.10	0.042 ^#^
G-aenial—Valux Plus	−13.20	0.003 ^#^	−10.10	0.041 ^#^	-	> 0.05
Gradia—Filtek Z550	-	> 0.05	-	> 0.05	-	> 0.05
Gradia—Valux Plus	-	> 0.05	-	> 0.05	-	> 0.05
Filtek Z550—Valux Plus	-	> 0.05	-	> 0.05	-	> 0.05
G-aenial—Valux Plus	-	> 0.05	-	> 0.05	-	> 0.05

* Tukey post hoc analysis. ** Games–Howell post hoc analysis. *** Dunn’s (1964) procedure with a Bonferroni correction. ^##^ 95% CI (lower to upper). ^#^ statistically significant (adjusted significance).

**Table 6:** Mean trace widths and friction coefficient measurements for all composites and protocols, sapphire ball.

Composite	GC G-Aenial Anterior		GC Gradia Direct Anterior		3M ESPE Filtek Z550		3M ESPE Valux Plus	
Trace Width								
Group	Trace width (median)	χ^2^(2)/ *p* *	Trace width (median)	χ^2^(2)/ *p* *	Trace width (median)	χ^2^(2)/ *p* *	Trace width (median)	χ^2^(2)/ *p* *
Control	215.21	2.540/ 0.281	216.59	10.519/ 0.005 ^#^	275.73	12.545/ 0.002 ^#^	242.62	7.819/ 0.020 ^#^
Home Bleach	225.45		234.03		298.58		278.65	
Office Bleach	225.97		247.49		305.50		289.68	
Friction Coefficient								
Group	Friction coeff (mean ± SD)	F(2,12)/ *p* **	Friction coeff (mean ± SD)	F(2,12)/ *p* **	Friction coeff (mean ± SD)	F(2,12)/ *p* **	Friction coeff (mean ± SD)	F(2,12)/ *p* **
Control	0.032 ± 0.0037	277.379/ 0.0005 ^#^	0.047 ± 0.0049	47.394/ 0.0005 ^#^	0.050 ± 0.0015	166.207/0.0005	0.041 ± 0.0025	77.368/0.0005 ^#^
Home Bleach	0.040 ± 0.0031		0.055 ± 0.0051		0.080 ± 0.0031		0.056 ± 0.0036	
Office Bleach	0.089 ± 0.0052		0.076 ± 0.0044		0.082 ± 0.0040		0.065 ± 0.0029	

* Kruskal–Wallis H test. ^#^ statistically significant. ** One-Way ANOVA.

**Table 7:** Trace widths and friction coefficients variation between groups, for all composites and protocols, sapphire ball—multiple group comparisons.

Composite	GC G-Aenial Anterior		GC Gradia Direct Anterior		3M ESPE Filtek Z550		3M ESPE Valux Plus	
Trace Width								
Group comparisons	Test statistic	*p* *	Test statistic	*p* *	Test statistic	*p* *	Test statistic	*p* *
Control—Home	Not applicable		-	> 0.05	-	> 0.05	-	> 0.05
Home—Office			-	> 0.05	-	> 0.05	-	> 0.05
Control—Office			−9.00	0.004 ^#^	−10.00	0.001 ^#^	−7.90	0.016
Friction Coefficient								
Group comparisons	Variation	*p* **	Variation	*p* **	Variation	*p* **	Variation	*p* **
Control—Home	0.008	0.025 ^#^	-	> 0.05	0.030	< 0.0005 ^#^	0.015	0.020 ^#^
Home—Office	0.049	< 0.0005 ^#^	0.021	< 0.0005 ^#^	-	> 0.05	0.009	0.002 ^#^
Control—Office	0.057	< 0.0005 ^#^	0.029	< 0.0005 ^#^	0.032	< 0.0005 ^#^	0.024	< 0.0005 ^#^

* Dunn’s (1964) procedure with a Bonferroni correction. ** Tukey post hoc analysis. ^#^ statistically significant (adjusted significance for trace widths).

**Table 8:** Trace widths and friction coefficient measurements for all composites and protocols, sapphire ball.

Composite	Control		Home Bleach		Office Bleach	
Trace Width						
Group	Trace width (median)	χ^2^(3)/ *p* *	Trace width (median)	χ^2^(3)/ *p* *	Trace width (median)	χ^2^(3)/ *p* *
GC G-aenial anterior	215.21	14.462/ 0.002 ^#^	225.45	16.727/ 0.001 ^#^	225.97	17.871/ < 0.0005 ^#^
GC Gradia direct anterior	216.59		234.03		247.49	
3M ESPE Filtek Z550	275.73		298.58		305.50	
3M ESPE Valux Plus	242.62		278.65		289.68	
Friction Coefficient						
Group	Friction coeff (mean ± SD)	F(3,8.263)/ *p* ***	Friction coeff (mean ± SD)	F(3,16)/ *p* **	Friction coeff (mean ± SD)	F(3,16)/ *p* **
GC G-aenial anterior	0.032 ± 0.0037	26.526/ < 0.0005 ^#^	0.040 ± 0.0031	91.194/ < 0.0005 ^#^	0.089 ± 0.0052	28.506/ < 0.0005 ^#^
GC Gradia direct anterior	0.047 ± 0.0049		0.055 ± 0.0051		0.076 ± 0.0044	
3M ESPE Filtek Z550	0.050 ± 0.0015		0.080 ± 0.0031		0.082 ± 0.0040	
3M ESPE Valux Plus	0.041 ± 0.0025		0.056 ± 0.0036		0.065 ± 0.0029	

* Kruskal–Wallis H test. ** One-Way ANOVA. *** Welch’s F. ^#^ statistically significant.

**Table 9:** Trace widths and friction coefficient variation between composites, for all protocols, sapphire ball—multiple group comparisons.

Composite	Control		Home Bleach		Office Bleach	
Trace Width						
Group comparisons	Test statistic	*p* *	Test statistic	*p* *	Test statistic	*p* *
G-aenial—GC Gradia	-	> 0.05	-	> 0.05	-	> 0.05
G-aenial—Filtek Z550	−11.40	0.014 ^#^	−14.00	0.001 ^#^	−15.00	< 0.0005 ^#^
G-aenial—Valux Plus	-	> 0.05	-	> 0.05	−10.00	0.045
Gradia—Filtek Z550	−11.40	0.014 ^#^	−11.00	0.020 ^#^	−10.00	0.045
Gradia—Valux Plus	-	> 0.05	-	> 0.05	-	> 0.05
Filtek Z550—Valux Plus	-	> 0.05	-	> 0.05	-	> 0.05
G-aenial—Valux Plus	-	> 0.05	-	> 0.05	-	> 0.05
Friction Coefficient						
Group comparisons	Variation	*p* ***	Variation	*p* **	Variation	*p* **
G-aenial—GC Gradia	0.015	0.024 ^#^	0.015	< 0.0005 ^#^	0.013	0.001 ^#^
G-aenial—Filtek Z550	0.018	< 0.0005 ^#^	0.040	< 0.0005 ^#^	-	> 0.05
G-aenial—Valux Plus	-	> 0.05	-	> 0.05	0.024	< 0.0005 ^#^
Gradia—Filtek Z550	-	> 0.05	-	> 0.05	-	> 0.05
Gradia—Valux Plus	-	> 0.05	-	> 0.05	0.011	0.004 ^#^
Filtek Z550—Valux Plus	0.009	0.001 ^#^	0.024	< 0.0005 ^#^	0.017	< 0.0005 ^#^
G-aenial—Valux Plus	-	> 0.05	-	> 0.05	-	> 0.05

* Dunn’s (1964) procedure with a Bonferroni correction. ** Tukey post hoc analysis. *** Games–Howell post hoc analysis. ^#^ statistically significant (adjusted significance for trace widths).

## Data Availability

The authors declare that the data of this research are available from the corresponding authors upon reasonable request.
